# Caregivers’ beliefs about hydroxyurea influence its usage among children with sickle cell disease: a cross-sectional study in Dar-es-Salaam, Tanzania

**DOI:** 10.11604/pamj.2025.52.31.45975

**Published:** 2025-09-19

**Authors:** Mwashungi Ally, Deodatus Kakoko, Calvin Swai, Tone Kristin Omsland, Kåre Moen, Emmy Metta, Mbonea Yonazi, Agnes Jonathan, Julie Makani, Elia John Mmbaga, Melkizedeck Leshabari, Emmanuel Balandya

**Affiliations:** 1 Department of Behavioral Sciences, Muhimbili University of Health and Allied Sciences, Dar-es-Salaam, Tanzania,; 2 Sickle Cell Program, Department of Haematology and Blood Transfusion, Muhimbili University of Health and Allied Sciences, Dar-es-Salaam, Tanzania,; 3 Department of Community Medicine and Global Health, University of Oslo, Oslo, Norway,; 4 Department of Educational Psychology and Curriculum Studies, University of Dodoma, Dodoma, Tanzania,; 5 Department of Haematology and Blood Transfusion, Muhimbili National Hospital, Dar-es-Salaam, Tanzania

**Keywords:** Sickle cell disease, hydroxyurea use, caregivers, beliefs about medications, perceived necessity, perceived concerns

## Abstract

**Introduction:**

hydroxyurea (HU) is a proven, safe, and effective treatment for sickle cell disease (SCD) but is underutilized in Tanzania. Caregivers´ beliefs influence medication usage in children with chronic diseases. This study assessed caregivers´ beliefs about HU and their effect on HU use among children with SCD in Dar-es-Salaam.

**Methods:**

a cross-sectional study enrolled 374 caregivers from four hospitals in Dar-es-Salaam. We adapted the “Belief About Medication Questionnaire” to assess caregivers´ beliefs about HU necessity and concerns. Scores of ≥15 indicated high necessity/concerns. Caregivers were categorized into four belief patterns: positive (high necessity, low concerns), negative (low necessity, high concerns), ambivalent (high necessity, high concerns), and indifferent (low necessity, low concerns).

**Results:**

seventy percent of caregivers reported HU usage, 63% exhibited positive beliefs, with 95% of them reporting HU use. Caregivers with an indifferent belief category made up 43% of those with non-positive beliefs, and only 7% of them reported HU use. Thirty-eight percent of caregivers were concerned about HU's long-term effects, whereas 67% perceived that HU protects their children´s health from worsening. Caregivers with positive beliefs were 14 times more likely to report HU use than those with indifferent HU beliefs (IRR 14.3, 95% CI: 5.5 - 36.9). Participants from national hospitals were three times more likely to have positive beliefs than those attending regional hospitals (aOR 3.3, 95% CI 2.1 - 5.1).

**Conclusion:**

health-care level and caregivers´ education impact HU beliefs and use. Targeted interventions to address specific obstacles across different caregiver subgroups are essential to improve HU beliefs and usage.

## Introduction

Sickle cell disease (SCD) is a life-threatening inherited blood disorder caused by a point mutation in the β-globin gene [1,2]. Over 300,000 babies with SCD are born every year, with over 75% living in sub-Saharan Africa (SSA), and the number is projected to rise to 404,200 births per year by 2050, with a corresponding increase in disease burden in SSA to 85% [2-5]. The prevalence of sickle cell trait is 20% in Tanzania, making it the fifth country with the highest burden of SCD globally [6-8]. Without comprehensive care, SCD is associated with high morbidity and mortality, with which 50% to 90% of children die before their fifth birthday [1,7,9,10]. Bone marrow transplant and gene therapy are the only curative options for SCD, but these are very expensive and not widely available in SSA [7,11]. Supportive care to prevent complications and manage symptoms of SCD remains the cornerstone of treatment in SSA. Hydroxyurea (HU) is the only proven safe and effective disease-modifying drug for the management of SCD available in SSA [7]. It was originally used for the treatment of hematological cancers and was first approved for use in SCD in the USA in 1998 [12,13]. Proper usage of HU has been shown to reduce the incidence of SCD complications and improve survival rates among patients with SCD [14-18]. Despite its safety and effectiveness, HU usage remains reportedly low in SSA [7,19-21]. In Tanzania, there are several ongoing efforts to increase HU usage in patients with SCD. These include the availability of HU in the National Medical Store Department, the incorporation of HU in the National Health Insurance Fund (NHIF), and the provision of free HU by the available SCD projects. However, the use of HU is still not optimal [7,19,20,22].

The use of medications is influenced by factors beyond the patients´ clinical and socio-demographic characteristics. Beliefs about medicines play an important role in medication use and adherence [23]. In this regard, a person´s perception of the necessity (benefits) and concerns (barriers) of medication use is pivotal in long-term usage and adherence to medication [24]. Beliefs toward certain medications are usually positive in cases where the perceived benefits of using such medication outweigh the perceived risks [23,25]. Studies have shown that beliefs about medications may offer greater predictability of medication use and medication adherence than clinical and social factors, and they vary in different areas depending on cultures, religions, and health literacy [26,27].

Studies from various countries have shown that perceived benefits such as reduced number of pain episodes, fewer blood transfusions and decreased hospital admissions, as well as concerns about HU side effects such as perceived risks of kidney disease, infertility, and weakened immunity, along with other perceived barriers to accessing HU, are associated with HU usage, adherence, and health-related quality of life among patients with SCD [22,28-32]. However, to the best of our knowledge, there are no data on the caregivers´ belief patterns about HU and how these beliefs may impact HU utilization among children with SCD under their care in Tanzania. This study assessed caregivers´ beliefs about hydroxyurea and their association with HU use among children with SCD attending public SCD clinics in Dar-es-Salaam, Tanzania.

## Methods

**Study design:** we conducted a hospital-based cross-sectional study using a quantitative approach for data collection.

**Study setting:** the study was conducted at four public SCD clinics in secondary and tertiary hospitals in the Dar-es-Salaam region for four months, from the 1^st^ of May 2023 to the 31^st^ of August 2023. These hospitals were Temeke and Amana Regional Referral Hospitals (RRH), the Muhimbili National Hospital (MNH), Upanga, and MNH Mloganzila. We excluded one regional hospital because HU was not available in the hospital during data collection. The SCD clinics at these facilities are among the clinical sites for the Sickle Pan-African Research Consortium (SPARCO)-Tanzania project. SPARCO-Tanzania has one of the largest databases of patients with SCD globally and is part of the SickleInAfrica Consortium [33]. These hospitals provide comprehensive care to patients with SCD, which includes screening and diagnosis of SCD, provision of routine SCD medications such as folic acid, hydroxyurea, and penicillin V, pain management, blood transfusion, health education, screening, prevention, and management of complications related to SCD.

**Study population and inclusion/exclusion criteria:** there are 4400 patients with SCD attending the selected hospitals and registered in the SPARCO database. Children under 18 years of age constitute 80% of all patients with SCD, and 65% of these children have health insurance subscriptions as reported in the SPARCO-Tanzania database. The majority of health-insured children have an NHIF subscription, which covers the cost of HU [20].

We used the formula for the calculation of sample size for a finite population to estimate the minimum sample size required to achieve 80% power of the study. We consecutively recruited 374 caregivers of health-insured SCD children under 18 years who sought healthcare services in the selected hospitals. Caregivers were adult males and females living with a child with SCD. The number of caregivers enrolled from each site reflected the proportion of health-insured children with SCD who attend these hospitals, as recorded in the SPARCO-Tanzania database, and ensured 80% power of the study. All children of caregivers enrolled in this study were registered in the SPARCO-Tanzania database with a confirmed diagnosis of SCD (HbSS). Caregivers of children who did not have a confirmed diagnosis of SCD, did not have health insurance coverage, or did not consent to participate in the study were excluded.

**Study variables:** independent variables included the caregiver´s age, sex, education level, occupation, caregiver-patient relationship, age of the child with SCD, level of the hospital where they attended. Dependent variables in our study were caregivers´ beliefs about HU and HU use among health-insured children with SCD. HU use was coded as 0 = no, 1=yes, where use meant current use or ever used HU in the past 12 months to minimize recall bias. Belief patterns were coded as 1 = positive beliefs, 2 = ambivalent, 3 = indifferent, and 4 = negative. Affordability and availability of HU were the potential confounders. To control the effect of these confounders, we enrolled caregivers of health-insured children with SCD attending public hospitals where HU was available and covered by health insurance. This ensured that caregivers could afford and access the medication.

**Data collection tools and procedures:** we trained four research assistants in preparation for data collection. The research assistants used the adapted standardized validated Belief about Medicine Questionnaire (BMQ) developed by Horne *et al*. to assess caregivers´ beliefs about HU [25,34]. The research assistants administered the questionnaires by face-to-face interviews in the waiting room where SCD clinics were conducted. The questionnaire consisted of concerns and necessity subscales, with 5 questions in each subscale, and scores for each question ranged from strongly disagree (1) to strongly agree (5). The necessity subscale assessed the perceived need to maintain the current and future health, while the concerns subscale assessed the perceived potential negative consequences associated with the use of prescribed medications. The scores ranged from 5 to 25. Participants who scored 15 and above were considered to have high scores [23,34,35]. Patients with high necessity and low concern scores were described as accepting the medicine (positive belief). In contrast, those with low necessity and high concern scores were termed as skeptical (negative belief). Patients with high necessity and high concerns were considered ambivalent, and those with low concerns and low necessity scores as indifferent [26,34,36]. We calculated the reliability of the adapted tools after data collection by assessing the internal consistency of the scales using Cronbach´s alpha. Both the necessity and concerns subscales had a reliability of Cronbach´s alpha of 0.9. One question was added to the questionnaire to inquire about the use of HU among children with SCD.

**Data management and analysis:** we kept the questionnaires safely in a locked cabinet accessed by the corresponding author. We inspected all questionnaires for completeness and consistency. All questionnaires were completely filled. We coded the data and entered it into the Statistical Package for Social Scientists (SPSS v29.0.1.0) software, which was used for data analysis. We summarized sociodemographic characteristics, beliefs, and HU usage as frequencies and proportions. We used a chi-square test to ascertain the association between caregivers´ socio-demographic characteristics and beliefs on HU. We used ordinal logistic regression to analyse factors associated with positive HU beliefs and modified Poisson regression for the association between caregivers´ beliefs and HU use among their children with SCD. Only variables with a p-value < 0.2 in univariable analysis were included in multivariable analysis. The differences in comparisons with a p-value of < 0.05 were considered statistically significant.

**Ethical approvals:** we obtained ethical approval from the institutional review boards at Muhimbili University of Health and Allied Sciences (MUHAS-REC-04-2023-1640) and the National Institution for Medical Research (NIMR/HQ/R.8a/Vol.IX/4409). All participants were adequately informed about the study, and they signed written informed consent before enrollment into the study. The participation of caregivers was performed in accordance with the Declaration of Helsinki.

## Results

**Socio-demographic characteristics of study participants:** we screened 393 caregivers before enrolment by inquiring about the age of their child who has SCD and their health insurance status. We excluded caregivers who did not have children with health insurance coverage or did not have confirmatory results for SCD. We enrolled a total of 374 caregivers of health-insured children with SCD. Most participants were women, comprising 86% of all caregivers. Sixty-five percent of caregivers were young adults below the age of 45 years. Forty-six percent had a primary education or less. Only 20% of caregivers had children with SCD aged above 13 years. Sixty-three percent were enrolled at the National hospitals, reflecting the proportion of insured children attending national hospitals as recorded in the SPARCO-Tanzania database (Table 1).

**Table 1:** socio-demographic and other characteristics of study participants

Variable		Frequency (%)
Caregivers' sex	Male	54 (14)
	Female	181 (86)
Caregivers’ age	< 35	102 (27)
	35 - 44	142 (38)
	≥ 45	130 (35)
Caregivers' education level	No formal education and primary education	173 (46)
	Secondary and higher education	201 (54)
Caregivers' occupation	Not employed	105 (28)
	Employed	269 (72)
Child's age group (years)	< 5	76 (20)
	5 - 12	224 (60)
	13 - 17	74 (20)
Caregiver-patient relationship	Parents	315 (84)
	Others	59 (16)
Level of health facility	Regional	138 (37)
	National	236 (63)

**Factors associated with caregivers´ beliefs about hydroxyurea:** seventy-four percent of caregivers had a high perceived necessity of HU use among their children with SCD. The median (IQR) score for perceived necessity was 20 (14 - 23). Sixty-seven percent of caregivers perceived that HU helps to protect their children´s health from deteriorating, 69% perceived that their children´s health at present depends on HU, 56% perceived that their children´s quality of health in the future depends on HU, 57% believed that without HU, their children´s entire life would be affected, and 61% perceived that without HU, their children would be very sick. On the other hand, the median score (IQR) for perceived concerns about HU was 9 (7 - 16). Overall, 28% of caregivers had high concerns about the use of HU among their children. The concerns were perceived fear of their children becoming dependent on HU (31%), perceived risk of long-term side effects associated with HU use (38%), worries that HU use interferes with their children daily lives (36%), concerns about their children taking HU on regular basis (28%) and HU being used for treatment of hematological cancers (37%). After combining the scores for both necessity and concerns subscales, 63% of caregivers were found to have positive HU beliefs, whereas 11% had ambivalent HU beliefs, 16% had indifferent HU beliefs, and 10% had negative HU beliefs. The proportion of those with positive HU beliefs was higher among those with secondary school education or higher than those with primary school education or lower (72% vs. 54%, p = 0.031). Further, the proportion of those with positive HU beliefs was higher among those attending National hospitals than among those attending the regional hospitals (73% vs. 46%, p = 0.001). The proportion of caregivers with positive HU beliefs was higher among caregivers aged 45 or older than those below 45 years (73% vs 54% and 61%, p = 0.001). Indifferent groups contributed the highest percentage among caregivers with unfavorable beliefs toward HU use, 43% of all non-positive beliefs. Sixty-two percent of caregivers with indifferent belief patterns had a primary education or less. Fifty-eight percent of caregivers with indifferent patterns attended regional hospitals (Table 2).

**Table 2:** factors associated with beliefs about hydroxyurea

Variable		Beliefs about hydroxyurea				P-value
		Negative (%)	Indifferent (%)	Ambivalent (%)	Positive (%)	
Caregivers' sex	Male	5 (9)	8 (15)	6 (11)	35 (65)	0.992
	Female	31 (10)	52 (16)	36 (11)	201 (63)	
Caregivers’ age	< 35	7 (7)	16 (16)	16 (16)	63 (61)	0.001
	35-44	15 (11)	26 (18)	24 (17)	77 (54)	
	≥ 45	14 (11)	18 (14)	2 (2)	96 (73)	
Caregivers' education level	No formal education and primary level	21 (12)	37 (21)	22 (13)	101(54)	0.031
	Secondary and higher education	15 (7)	23 (11)	20 (10)	135 (72)	
Caregivers' occupation	Not employed	6 (6)	14 (13)	11 (11)	74 (71)	0.229
	Employed	30 (11)	46 (17)	31 (12)	162 (60)	
Child's age group (years)	< 5	8 (11)	15 (20)	10 (13)	43 (57)	0.432
	5 - 12	21 (9)	36 (16)	28 (13)	139 (62)	
	13 - 17	7 (10)	9 (12)	4 (5)	54 (73)	
Caregiver-patient relationship	Parents	27 (9)	53 (17)	37 (12)	198 (63)	0.313
	Others	9 (15)	7 (12)	5 (9)	38 (64)	
Level of health facility	Regional	19 (14)	35 (25)	21 (15)	63 (46)	0.001
	National	17 (7)	25 (11)	21 (9)	173 (73)	

**Association between caregivers´ beliefs about HU and utilization of HU among children with SCD:** seventy percent of caregivers reported HU use among their children with SCD. Ninety-five percent (225/236) of caregivers with positive HU beliefs were giving HU to their children, whereas only 7% (4/60), 33% (12/36), and 52% (22/42) of children whose caregivers had indifferent, negative, and ambivalent HU belief patterns were using HU. The differences in HU use among caregivers with different HU beliefs were statistically significant, with a p-value of 0.001 (Table 3).

**Table 3:** association between caregivers' belief patterns and hydroxyurea use

Variable	Category	Hydroxyurea use among children with SCD		IRR	95% CI
		No n (%)	Yes n (%)		
Caregivers' hydroxyurea belief patterns	Positive HU belief	11 (5)	225 (95)	14.3	5.5 - 36.9
	Ambivalent HU belief	20 (48)	22 (52)	7.9	2.9 - 21.2
	Negative HU belief	24 (67)	12 (33)	5.0	1.7 - 14.4
	Indifferent HU belief	56 (93)	4 (7)	Ref	

IRR: incidence rate ratio; Ref: reference category; HU: hydroxyurea; SCD: sickle cell disease

Caregivers with indifferent HU beliefs reported the lowest proportion of children using HU (7%). Caregivers with positive HU beliefs were 14 times more likely to give HU to their children than those with indifferent HU beliefs (incidence rate ratio; IRR 14.3, 95% CI 5.5 - 36.9). Caregivers with negative HU beliefs were 5 times more likely to give HU to their children than those with indifferent HU beliefs (incidence rate ratio; IRR 5.0, 95% CI 1.7 - 14.4). Further, caregivers with ambivalent beliefs were 8 times more likely to give HU to their children compared with caregivers with indifferent beliefs (IRR 7.9, 95% CI 2.9 - 21.2) (Table 3).

**Factors associated with positive beliefs about hydroxyurea:** age of the child, caregiver´s education level, and level of hospital had a p-value of < 0.2 in univariable analysis, and therefore, they were included in multivariable analysis. After adjusting for the child´s age, the caregiver´s education, and level of hospital in the multivariable analysis, caregivers with at least secondary education were 2.3 times more likely to have positive HU beliefs compared to other belief patterns than those with primary education or less (aOR 2.3, 95% CI 1.5 - 3.5). Caregivers of children under 5 years were less likely to hold positive HU beliefs compared to other belief patterns than those with children above 13 years (aOR 0.4, 95% CI 0.2 - 0.8). Additionally, caregivers attending National hospitals were 3.3 times more likely to have positive HU beliefs compared to other belief patterns than those attending referral hospitals (aOR 3.3, 95% CI 2.1 - 5.1). Unadjusted estimates are shown in Table 4.

**Table 4:** factors associated with positive beliefs about hydroxyurea

Variable	Category	Univariable analysis		Multivariable analysis	
		cOR	95% CI	aOR	95% CI
Caregivers' sex	Female	0.9	0.5 - 1.6		
	Male	Ref			
Caregivers’ age	35-44	0.7	0.4 - 1.2		
	≥ 45	1.4	0.8 - 2.5		
	< 35	Ref			
Caregivers' education level	Secondary and higher education	1.8	1.2 - 2.8	2.3	1.5 - 3.5
	No formal education and primary level	Ref			
Caregivers' occupation	Employed	0.6	0.4 - 0.9	1.4	0.8 - 2.2
	Unemployed	Ref			
Child's age group (years)	< 5	0.5	0.3 - 1.1	0.4	0.2 - 0.8
	5 - 12	0.7	0.4 - 1.2	0.5	0.3 - 1.1
	13 - 17	Ref			
Caregiver-patient relationship	Parents	1.1	0.6 - 1.8		
	Others	Ref			
Level of health facility	National	3.0	2.0 - 4.7	3.3	2.1 - 5.1
	Regional	Ref			

cOR: crude odds ratio; aOR: adjusted odds ratio; Ref: reference category; odds ratios were derived from ordinal logistic regression with outcome variable following the following order: negative HU belief | indifferent HU belief | ambivalent HU belief | positive HU belief; HU: hydroxyurea

## Discussion

Although various studies have reported the impact of beliefs about medications in the management of patients with chronic diseases, this is the first study to describe caregivers´ belief patterns towards the use of HU, and report its effect on HU use in children with SCD in Tanzania.

Our study found a strong positive association between caregivers´ positive HU beliefs and HU use among their children with SCD (IRR 14.3, 95% CI 5.5 - 36.9). This is similar to other studies showing that individuals with positive medication beliefs demonstrate high use and adherence to medicines [27,34,35]. Positive beliefs about medication indicate that an individual has a strong sense of personal need for the medication to maintain their current and future health. In contrast, negative beliefs reflect heightened concerns about the potential negative effects of the medication, hence reducing the likelihood of using the prescribed medication [23]. We noted that caregivers with low perceptions of HU necessity combined with low concerns about HU use (indifferent pattern) reported the lowest HU usage among their children (7%). The difference may be attributed to the lower education levels observed among caregivers in the indifferent group, making it difficult for them to understand about benefits and potential concerns of HU use. Three-quarters of caregivers had a high perceived necessity of HU use, indicating their belief that HU is necessary for the current and future well-being of their children with SCD. Other studies have shown similar findings that the majority of caregivers of children with SCD reported a high perceived necessity of HU use in maintaining the health of their children since HU use is associated with reduced incidence of complications and hospital visits [30]. In our study, caregivers were concerned about their children becoming dependent on HU (31%), perceived fear of long-term side effects specifically on fertility (38%), worries that HU use interferes with their children daily lives (36%), concerns about their children taking HU on regular basis (28%) and HU being a drug used for treatment of hematological cancers (37%). Daily use of medications and perceived risk of long-term side effects have been mentioned in other studies as the major concerns among parents and patients with chronic diseases [22,24,37,38]. Customized efforts should be directed to each subgroup to enhance caregivers´ understanding of SCD, safety, benefits, and potential side effects associated with HU use.

We observed that caregivers with primary education or less comprised 46% of caregivers with negative, indifferent, and ambivalent HU beliefs, whereas 72% of those with secondary and higher education had positive beliefs. Caregivers with at least secondary education were 2.3 times more likely to have positive beliefs than those with primary education or without formal education. Further, 62% of caregivers with indifferent HU beliefs had primary education or less, and this group contributed the highest percentage of caregivers with unfavorable beliefs towards HU use. This is similar to findings from other studies showing that an increased level of education is associated with improved beliefs about medications [39,40]. Individuals with lower education levels are also more likely to seek care from traditional or spiritual healers [41]. A higher level of education is associated with increased health information-seeking behaviors and health literacy, leading to a higher level of understanding of benefits, mechanisms of action, side effects of the medicines, and ways to reduce perceived barriers to medication usage [39,40,42-44]. Therefore, there is a need to assess different approaches to providing health information to caregivers with low education levels to maximize their understanding of the benefits and adverse effects of HU.

In our study, caregivers attending national hospitals (MNH Upanga and Mloganzila), the tertiary referral hospitals in Tanzania, were more likely to have positive beliefs compared to other belief categories. In contrast, 58% of caregivers with indifferent belief patterns, the group that reported the lowest proportion of children using HU, attended the lower-level hospitals. Although there were no statistically significant differences in education level and occupation among caregivers attending these hospitals, most caregivers at National hospitals had secondary or higher education (56%), whereas the majority of those at regional hospitals had only primary education or no formal education (54%), with a p-value of 0.07. Studies suggest that individuals with higher education levels are more likely to seek care at higher-level health facilities [45,46]. Individuals attending lower-level hospitals are likely to live closer to hospitals and have lower education levels [45]. Additionally, in a study conducted in 2021 in similar facilities, only 25% of healthcare providers in regional referral hospitals in Dar-es-Salaam were found to have adequate knowledge of SCD and its management [47]. Therefore, the differences in HU beliefs among caregivers may be due to variations in the quantity and quality of health education on HU provided in these hospitals, SCD knowledge, and the level of understanding of health information among caregivers. Health education has been found to improve caregivers' and patients´ understanding and perceptions of disease prevention and management [39,48].

Additionally, we observed that caregivers of children under five years were less likely to have positive HU beliefs compared to other belief patterns than those with older children (aOR 0.4, 95% CI 0.2 - 0.8). In patients with inherited chronic diseases, older age is often associated with increased likelihood of complications and greater caregiver understanding of the benefits and side effects of prescribed medications. It is known that reduced frequency of SCD complications, fewer admissions, and improved quality of life after the use of HU are associated with high perceived necessity of HU among parents of children with SCD [30,38]. Therefore, a smaller number of admissions and complications experienced by caregivers of younger children may have contributed to the differences observed in HU belief patterns among caregivers of children younger than 5 years and those with children above 12 years. Even though we did not assess difficulties in administering HU in this study, the absence of a HU pediatric formulation has been mentioned as one of the caregivers´ concerns regarding HU use in children under five years [21,49].

**Study strength and limitations:** a key strength of this study was the inclusion of a large number of caregivers of health-insured children with SCD who attended public SCD clinics in Dar-es-Salaam, where hydroxyurea was accessible. This enabled us to address caregivers´ perceived concerns and benefits regarding HU use and study the potential effects of caregivers´ beliefs on the usage of HU among children, an often-forgotten interface, while controlling for the ability to obtain HU through health insurance, which is typically a major limiting factor for accessing HU. Notwithstanding this strength, our study was limited by the inability to explore caregivers´ knowledge of HU, caregivers´ perceived barriers in accessing HU, and engagement in shared decision-making, which could also explain their beliefs about HU. Consecutive sampling technique and exclusion of caregivers of uninsured children could limit generalizability; therefore, the findings of this study could be applicable in settings where HU is accessible to patients with SCD.

## Conclusion

About two-thirds of caregivers of children with SCD in Dar-es-Salaam had positive beliefs about HU. Three-quarters of caregivers had a high perceived necessity of HU use, indicating their belief that HU is necessary for the current and future well-being of their children with SCD. Over one-third of caregivers are concerned about the safety of long-term use of HU. Caregivers´ education level and the level of the healthcare facility where care is provided were associated with positive HU beliefs and, subsequently, HU usage among children with SCD. Caregivers of children under five years are less likely to have positive HU beliefs and give HU to their children. To accelerate HU use among children with SCD, targeted interventions should be implemented for each subgroup. These may include engaging caregivers in SCD support groups, providing tailored health education programs for caregivers with lower education levels and caregivers of children under five years, and capacitating healthcare workers in lower-level hospitals to counsel and educate caregivers about HU. This approach would help to increase perceived necessity and address perceived barriers to HU use, complementing the ongoing interventions to increase HU use among children with SCD in Tanzania.

### What is known about this topic

- The limited use of HU remains a significant challenge to optimizing care for individuals with SCD in SSA;
- Perceived benefits (necessity) and perceived barriers (concerns), and beliefs about medication impact the use of medications in patients with chronic diseases.

### What this study adds

- We demonstrate the first evidence from Tanzania that caregivers' belief patterns about HU impact its usage among their children with SCD, even when they have unrestricted access through health insurance and HU availability in the hospitals;
- Additionally, we found that caregivers´ low education level and receiving care at lower-level healthcare facilities were associated with unfavorable HU beliefs and, subsequently, low HU usage among children with SCD;
- These findings underscore the importance of scaling up tailored education interventions in lower-level health facilities to improve caregivers´ beliefs about HU and accelerate its usage among children with SCD in SSA.
